# Genome-Wide Identification, Expression Analysis, and Subcellular Localization of *DET2* Gene Family in *Populus yunnanensis*

**DOI:** 10.3390/genes15020148

**Published:** 2024-01-23

**Authors:** Zhensheng Qiao, Jiaqi Li, Xiaolin Zhang, Haiyang Guo, Chengzhong He, Dan Zong

**Affiliations:** 1 College of Life Sciences, Southwest Forestry University, Kunming 650224, China; ipssqzs@swfu.edu.cn (Z.Q.); lijiaqi@swfu.edu.cn (J.L.); ghy10101205@163.com (H.G.); hecz@swfu.edu.cn (C.H.); 2 Key Laboratory for Forest Genetic and Tree Improvement and Propagation in University of YunnanProvince, Southwest Forestry University, Kunming 650224, China; sunnyxlz27@163.com; 3 College of Forestry, Southwest Forestry University, Kunming 650224, China; 4 Key Laboratory of Biodiversity Conservation in Southwest China, State Forestry Administration, Southwest Forestry University, Kunming 650224, China

**Keywords:** *Populus yunnanensis*, *DET2*, subcellular localization, brassinolides biosynthesis, rapid growth

## Abstract

(1) Background: Brassinosteroids (BRs) are important hormones involved in almost all stages of plant growth and development, and sterol dehydrogenase is a key enzyme involved in BRs biosynthesis. However, the sterol dehydrogenase gene family of *Populus yunnanensis* Dode (*P. yunnanensis*) has not been studied. (2) Methods: The *PyDET2* (*DEETIOLATED2*) gene family was identified and analyzed. Three genes were screened based on RNA-seq of the stem tips, and the *PyDET2e* was further investigated via qRT-PCR (quantitative real-time polymerase chain reaction) and subcellular localization. (3) Results: The 14 *DET2* family genes in *P. yunnanensis* were categorized into four groups, and 10 conserved protein motifs were identified. The gene structure, chromosome distribution, collinearity, and codon preference of all *PyDET2* genes in the *P. yunnanensis* genome were analyzed. The codon preference of this family is towards the A/U ending, which is strongly influenced by natural selection. The *PyDET2e* gene was expressed at a higher level in September than in July, and it was significantly expressed in stems, stem tips, and leaves. The *PyDET2e* protein was localized in chloroplasts. (4) Conclusions: The *PyDET2e* plays an important role in the rapid growth period of *P. yunnanensis*. This systematic analysis provides a basis for the genome-wide identification of genes related to the brassinolide biosynthesis process in *P. yunnanensis*, and lays a foundation for the study of the rapid growth mechanism of *P. yunnanensis*.

## 1. Introduction

Brassinosteroids (BRs) are the sixth natural plant hormones after auxin, gibberellin, cytokinin, abscisic acid, and ethylene, which can play a positive role in plant growth and development [1], senescence process [2], nutrient accumulation and substance synthesis [3,4], and plant stress tolerance in plant [5,6,7,8].

There are three BR synthesis pathways in plants: the early C-6 oxidation pathway and the late C-6 oxidation pathway, which are dependent on canberylalcohol (CN), and the early C-22 and C-23 hydroxylation pathways, which are CN-independent [9]. Based on the previous studies of *Arabidopsis thaliana*, the CN-dependent pathway is the main way of BR biosynthesis [10], and multiple genes were identified, such as *DWF4* (*DWARF4)* [11], *BBX21* (*B-BOX-CONTAINING ZINC FINGER TRANSCRIPTION FACTOR 21*) [12], *CPD* (*CONSTITUTIVE PHOTOMORPOGENIC DWARF*) [13], *CYP90C1* (*CYTOCHROME P450 90C1*) and *CYP90D1* (*CYTOCHROME P450 90D1*) [14,15], *BR6ox1/2* (*BRASSINOSTEROID-6-OXIDASE1/2*) [16], *DET2* (*DEETIOLATED2)* [17], and so on.

The *DET2* gene of *A. thaliana*, known as the *DWF6* gene, is found in *det2* (*deetiolated2*) mutants and is highly consistent with steroid 5α-reductase, which catalyzes the synthesis of dihydrotestosterone in animals and catalyzes the development of the male genitalia and prostate during animal embryonic development [18]. The *DET2 gene* in plants is the rate-limiting gene in three pathways of BRs biosynthesis, and all the intermediates in the BR biosynthetic pathway after the *DET2* reaction can be used to rescue the *det2* mutant phenotype [19]. Many studies have speculated that the *DET2* gene affects cell division and growth during plant development by regulating BR content and then regulating cell wall synthesis and extension. The expression of *CycD3* was significantly increased after treatment with 24-epibrassinolide (24-EBL) in *A. thaliana*, which indicated that brassinolide might have some effect on cell division, and the in vitro application of brassinolide to the *DET2* mutant of *A. thaliana* could increase the number and size of its leaf cells [20]. The study of BR-related mutants (*det2-1* and *bril-301*) in *A. thaliana* showed the expression of cellulose synthase gene and the content of cellulose decreased significantly [21]. When the amino acid Glu240 in the *DET2* protein was substituted, the activity of 5α-dehydrogenase was completely lost, resulting in a decrease in BR biosynthesis, the mutant cells reduced, and the plant appeared yellowing and dwarfing [22].The over expression of the *DET2* gene showed the elongation of fibroblasts [23], the improvement of endogenous castasterone (CS) levels, and the enhancement of cell activity in cambium meristem and xylem (XY) differentiation to promote stem growth [24,25].

The fast growth of forest trees is determined by the comprehensive action of many factors of external and endogenous signals [26,27]. As a representative species of *Populus* in China, *Populus yunnanensis* is adaptable, fast-growing, and easily survives through cuttings. It is primarily distributed in mountainous regions ranging from 1600 to 3200 m in Southwest China. So it plays an important role in forestry production, ecological protection, industrial raw materials, and building materials [28]. Because of its stronger photosynthetic ability and tissue-life ability than other poplar species, it grows more rapidly. The peak of plant height growth occurs from June to October. Even at altitudes of 3000 to 4000 m, the tree can achieve a height growth of 1.0 to 2.4 m, demonstrating robust and rapid development, along with a significant value for utilization [29].

In the organs, tissues, or cells of plants, there are some morphological and biochemical gradients in different axes, which is called polarity. Polarity is a basic phenomenon in plant differentiation. Once it is established, the growth and development of plants have a specific direction, and it is difficult to reverse. Previous studies have shown that upright and inverted cuttings of *P. yunnanensis* could survive, and both grew faster in September, so their height and stem diameter were significantly higher than those in July. In this study, the steroidal dehydrogenase family of *P. yunnanensis* was identified and other bioinformatics were analyzed. The growth of the stem is caused by the continuous or periodic cell division of the meristem at the end of the stem, which makes the stem grow. The differentially expressed genes of this family were screened based on the transcriptome of the stem tip, and the expression and subcellular localization were analyzed to elucidate the molecular mechanism of *PyDET2e* involved in the rapid growth of *P. yunnanensis*.

## 2. Materials and Methods

### 2.1. Plant Materials and Vector

The cuttings of 3 clones of *P. yunnanensis* were cultivated in the greenhouse for one year (Southwest Forestry University, Kunming, China. 102.76 E, 25.06 N). In February 2022, we obtained about 15 cm cuttings and preserved these in the soil. The cuttings with different tissues were used in experiments performed in July 2023. We obtained roots, stem, lateral buds, branches, young leaves (1st to 3rd leaves counted from the stem tip), and the stem tip. All plant samples collected were brought back in liquid nitrogen and then stored at −80°C until the next use. The plant expression vector pSuper1300-*GFP* was kept in our laboratory.

### 2.2. Date Sources

The complete genome sequence and annotation files of *P. yunnanensis* wereretrieved from our laboratory (Southwest Forestry University, Kunming 6500224, China) (BioProject:PRJNA886471).The RNA-seq data used in this study were obtained from previous studies in our laboratory. The *DET2* protein sequences of *A. thaliana* and *P. trichocarpa* were obtained from the phytozome database (https://phytozome-next.jgi.doe.gov/, accessed on 15 June 2023).

### 2.3. Genome-Wide Identification of DET2 Gene Members in P. yunnanensis

The HMM data of the steroid dehydrogenase structural domain (PF02544) were obtained from the online server of the Inter Pro database (https://www.ebi.ac.uk/interpro/, accessed on 15 June 2023). Then, the protein sequences of the *DET2* gene family members of *A. thaliana* and *P. trichocarpa* were downloaded from the Phytozome database (https://phytozome-next.jgi.doe.gov/, accessed on 15 June 2023) using PF02544. Proteome files were extracted using TBtools (v2.008) based on the genome and GFF files of *P. yunnanensis* [30]. First, we queried the homologous sequences in the *P. yunnanensis* genome based on the known *AtDET2* protein sequences by performing a BLASTp search (E-value < 10^−14^) with TBtools (v2.008). Then, we further screened the conserved structural domains using the NCBI Conserved Structural Domain Search tool (CD-Search) (https://www.ncbi.nlm.nih.gov/Structure/cdd/wrpsb.cgi, accessed on 15 June 2023). After removing incomplete and redundant sequences, the existence of the *DET2* gene family of *P. yunnanensis* was determined.

### 2.4. Amino Acid Sequence and Phylogenetic Analysis

The Expasy website (http://web.expasy.org/protParam/, accessed on 15 June 2023) was used to analyze the physical and chemical properties of family members; the Subcellular localization predictions and secondary structure predictions were performed by the PSORT website (https://www.genscript.com/psort.html, accessed on 15 June 2023) and the Spoma website (https://npsa-pbil.ibcp.fr/cgi-bin/npsa_automat.pl?page=npsa_sopma.html, accessed on 15 June 2023). We used MEGA11 software (v11.0.13) for evolutionary tree construction [31]. Multiple comparisons of amino acid sequences were performed using ClustalW.

### 2.5. Gene Structure, Conserved Motif, and Cis-Acting Element Analysis

The conserved motifs of the *DET2* proteins were analyzed using the MEME Suite tool (https://meme-suite.org/meme/tools/meme, accessed on 16 June 2023). The 2.0 kb sequence upstream of each *DET2* gene was retrieved and submitted to the PlantCARE website (http://bioinformatics.psb.ugent.be/webtools//plantcare/html/, accessed on 16 June 2023). The conserved structural domains, gene structures, motifs, and cis-elements of the *DET2* proteins in the *DET2* genes were visualized using TBtools (v2.008).

### 2.6. Chromosomal Location and Collinearity Analysis

The location of *PyDET2s* genes on chromosomes and collinearity analysis were visualized using TBtools (v2.008).

### 2.7. Codon Bias and Influence Factors Analysis

CodonW and Emboss (http://emboss.toulouse.inra.fr/cgi-bin/emboss/chips, accessed on 17 June 2023) were used to calculate the codon bias parameters of the coding proteins of the *DET2* gene family of *P. yunnanensis*, and then the analysis ENc-Plot, PR2-plot, and Neutral mapping was conducted using R Studio (V3.6.0) [32].

### 2.8. Expression Pattern Analysis of PyDET2e

Expression patterns were analyzed using TBtools software by constructing *PyDET2s* expression heatmaps based on RNA-seq data. The primers for qRT-PCR were designed from CDS sequences using the NCBI primer design tool (https://www.ncbi.nlm.nih.gov/tools/primer-blast/, accessed on 19 June 2023) (Table S1).

Firstly, the total RNA extraction was performed using the E.Z.N.A@ Plant RNA kit (Omega Bio-tek Inc., Beijing, China), and the quality and purity of RNA were detected using K5800C (KAIAO, Beijing, China). The 1-st cDNA synthesis was performed using Hifair^®^ III Reverse Transcriptase (YEASEN, Shanghai, China) on 500ng of RNA from each sample. The cDNA was then diluted 7-fold for qRT-PCR. The Applied Biosystems 6800 real-time PCR machine and Hieff SYBR Green Master Mix (Yeasen Biotechnology Co. Ltd., Shanghai, China) were used to perform qRT-PCR following the specified system and procedures. The reaction system (20.0 µL) included the following: 1.0 µL cDNA template, 0.4 µM primer (F/R), 10 µL mix, and 8.2 µL RNase-free water. The qRT-PCR thermal cycle conditions were as follows: denaturation at 95 °C (2 min), 45 cycles at 95 °C (10 s), and 56 °C (30 s). Fluorescence intensities were measured for qRT-PCR at the end of each cycle. The relative transcript abundance values were calculated using the 2^−∆∆Ct^ method. The data statistical analysis and visualization were performed by SPSS21 (*p*-values equal to 0.05 were kept statistically significant) and GraphPad (v8.0.2).

### 2.9. Subcellular Localization of PyDET2e

The localization of *PyDET2e* was determined by injecting tobacco epidermal cells using the pSuper1300-*GFP* vector with the CaMV35S promoter. For this purpose, we designed appropriate primers with restriction sites (*Xbal 1* and *Hind III*) according to the CDS sequence of *PyDET2e* and the multiple cloning site sequence in the vector and cloned this fragment into the pMD-19T vector. Then, positive bacteria were sent for sequencing (Shanghai Biotechnology Co., Ltd., Shanghai, China) after transformation. After sequencing, the gene fragments were digested with the restriction enzymes (*Xbal 1* and *Hind III*) and ligated into the pSuper1300-*GFP* vector digested by *Xbal 1 and Hind III* using T4 ligase (Takara Bio, Shanghai, China). The over expression vector *PyDET2e*-pSuper1300-*GFP* was successfully transformed into tobacco epidermal cells, while pSuper1300-*GFP* alone was transformed to use as a control. Three days after injection into the abaxial epidermis of tobacco leaves, protein expression was observed using fluorescence confocal microscopy.

## 3. Results

### 3.1. Identification of the DET2 Genes in P. yunnanensis

Fourteen *DET2* genes were identified in *P. yunnanensis* and named *PyDET2a*-*PyDET2n* based on their positions on the chromosome (Table S2). They differed in sequence length, isoelectric point, and molecular weight. The amino acid length variedfrom 253 aa (*PyDET2b*) to 351 aa (*PyDET2f*). The molecular weight ranged from 20.71 kDa (*PyDET2k*) to 40.35 kDa (*PyDET2f*) with an average of 31.44 kDa, and the pI ranged from 8.79 (*PyDET2a*) to 9.66 (*PyDET2e*) with an average of 9.32. The analysis of the total average index of hydrophobicity (GRAVY) showed that all proteins were hydrophobic. Most of the proteins were unstable with an instability index ranging from 22.63 (*PyDET2l*) to 46.84 (*PyDET2a*). The secondary structure of all proteins was dominated by α-helices (Table S2 and Figure S1). Subcellular localization predictions indicated that most were mainly located in chloroplasts and cell membranes.

### 3.2. Phylogenetic Analysis and Motif Elicitation of PyDET2s

**Figure 1:**
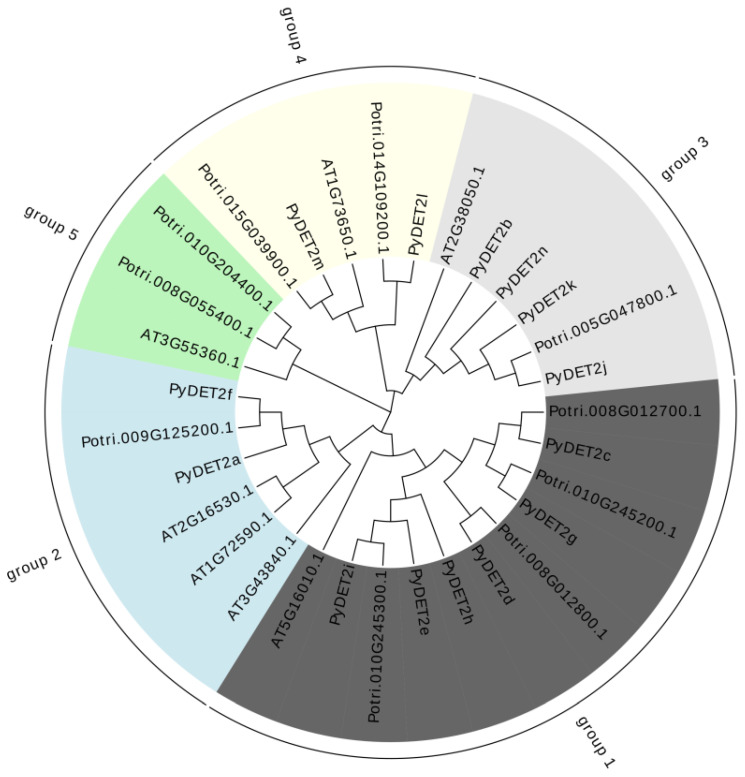
Phylogenetic tree of 31 *DET2* proteins from three species. Five subgroups (group 1, group 2, group 3, group 4, and group 5) are shown in different colors.

**Figure 2:**
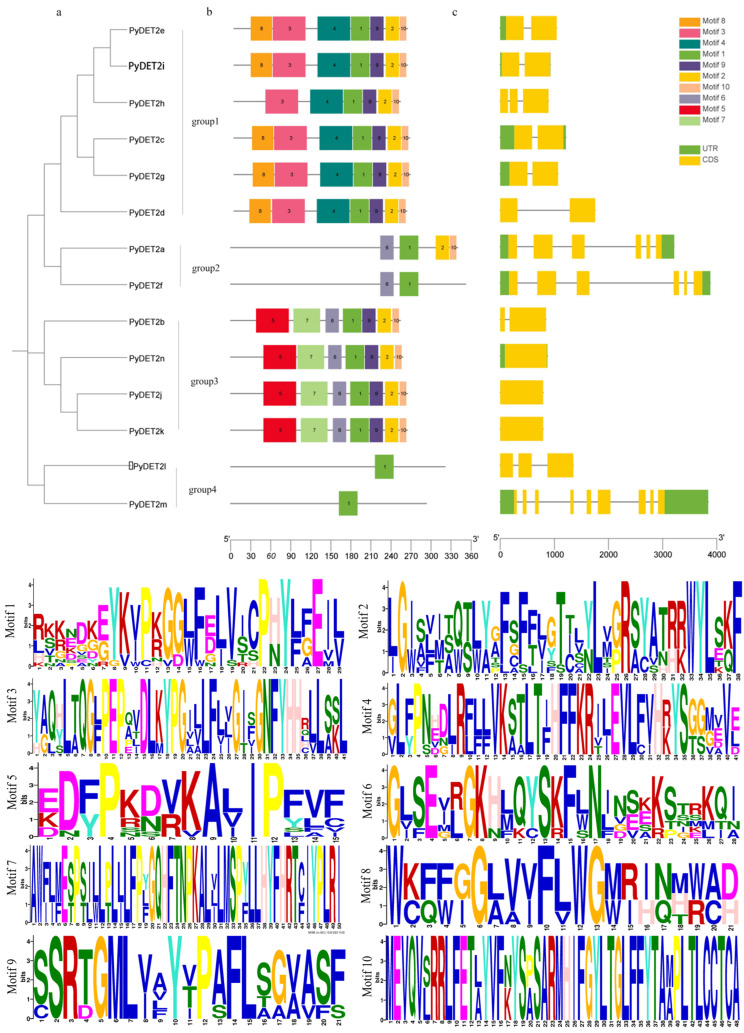
The conserved motifs of proteins and gene structure depending on the phylogenetic relationships of *DET2* genes in *P. yunnanensis*. (**a**) The phylogenetic tree of 14 *DET2* proteins was built. Four subgroups (group 1, group2, group 3, and group 4) are shown in different colors. (**b**) Analysis of conserved motifs of *DET2* genes in *P. yunnanensis*. Gray lines represent sequences of different lengths, and blocks of different colors represent different conserved motifs. (**c**) Exon and intron structure analysis of *DET2* genes in *P. yunnanensis*. Black lines represent introns, yellow boxes represent exons, and green boxes represent untranslated regions (UTR).

The phylogenetic tree to understand the evolutionary relationships of three species using the protein sequences of the members was built, which consisted of 7 *AtDET2s*, 14 *PyDET2s*, and 10 *PtDET2s*, which were unevenly divided into five subgroups (Figure 1). We also found that the genes in the same group had similar structures (Figure 2).

The conserved protein motifs of the *DET2* proteins are shown in Figure 2. It was found that approximately all sequences contained a conserved protein motif with a width of 29 amino acids, which appears as a dark green block (motif 1), which is presumed to be conservative in this family, and this motif was present at the N-terminus of all members. The composition of the motif is similar in the same subgroup. The first subgroup contained motif 1, 2, 3, 4, 9, and 10. The second subgroup contained motif 9 and 10. The third subgroup contained motif 1, 2, 5, 6, 7, 9, and 10. The fourth subgroup only contained motif 1.

### 3.3. Localization and Duplication of PyDET2s

**Figure 3:**
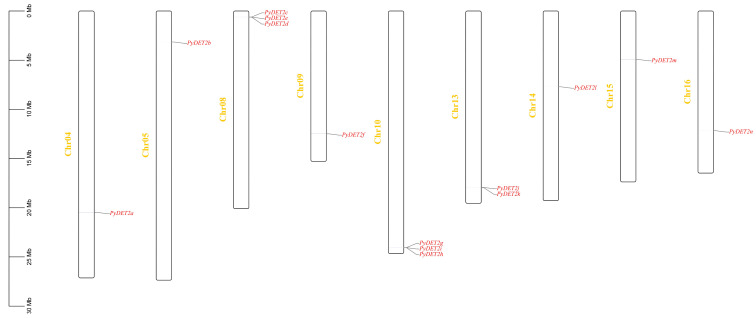
The distribution of *PyDET2s* in chromosomes. The vertical bar represents the chromosomes of *P. yunnanensis*. The scale on the left indicates chromosome length. From left to right are 9 chromosomes with *DET2* gene distribution. *The PyDET2s* are shown in red.

The result of chromosome localization is shown in Figure 3. Fourteen members were distributed in chromosome 4, 5, 8, 9, 10, 13, 14, and 15 and chromosome 16, and there were serial replications in chromosome 8 (*PyDET2c*, *PyDET2e*, and *PyDET2d*), chromosome 10 (*PyDET2g*, *PyDET2i*, and *PyDET2h*), and chromosome 13 (*PyDET2j* and *PyDET2k*).

**Figure 4:**
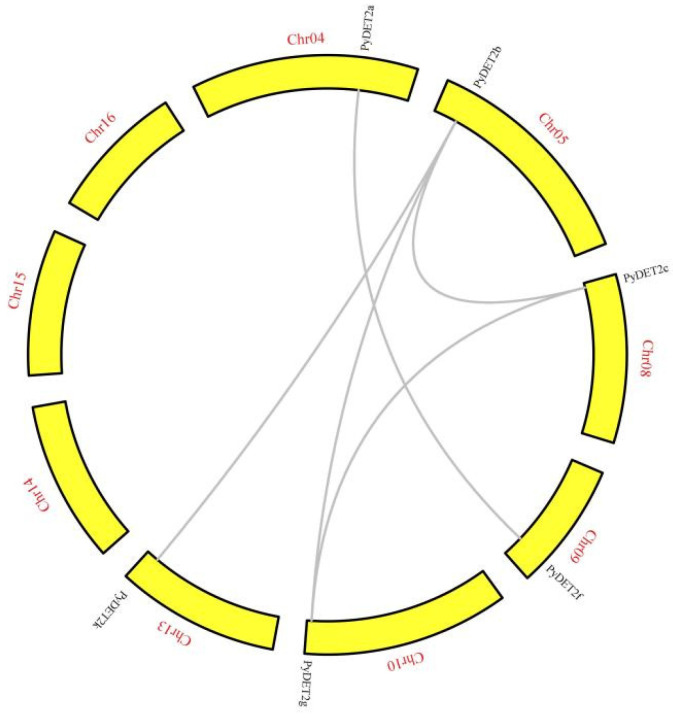
Synteny analysis of *DET2* family members in *P. yunnanensis*. The genes linked by the gray line represent the homologous *DET2s* between *PyDET2s*.

**Figure 5:**
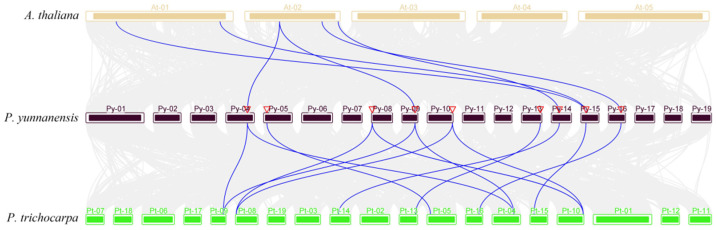
Collinearity analysis of *DET2s* among three species. All collinear genes were labeled in gray, while the collinear *DET2* gene pairs were labeled in blue.

We analyzed the collinear blocks and gene duplication types. Five duplication *PyDET2* gene pairs were observed (*PyDET2a*-*PyDET2f*, *PyDET2b*-*PyDET2c*, *PyDET2b*-*PyDET2g*, *PyDET2b*-*PyDET2j*, and *PyDET2c*-*PyDET2g*), and located on different chromosomes (chromosome 4, 5, 8, 9, 10, and 13) in *P. yunnanensis* (Figure 4). During the evolution of *PyDET2s*, the replication events including whole genome duplication and segmental duplication were identified. A total of 6 pairs of collinearity genes were found between *P. yunnanensis* and *A. Thaliana*, and 14 pairs of collinearity genes were found between *P. yunnanensis* and *P. trichocarpa*, but no collinearity was found between *PyDET2d*, *h*, *i* and *k* (Figure 5).

### 3.4. Prediction of Cis-Regulatory Elements in the Promoters of PyDET2s

**Figure 6:**
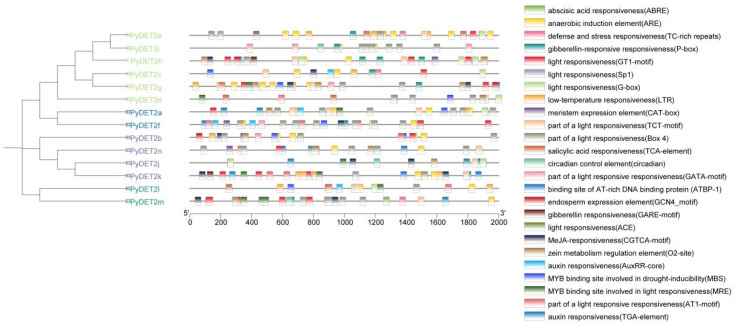
Cis-acting elements in the promoter regions of 14 *DET2* genes. The black lines represent the promoter length. The different colored boxes represent different elements.

We studied the potential regulation of cis-acting elements in *PyDET2s* (Figure 6). Twenty-five cis-regulatory elements were identified, and light responsiveness was the most important element. We also found many hormone responses, and stress elements were abundant in the promoter regions of family members, such as abscisic acid responsiveness (ABRE), anaerobic induction element (ARE), MeJA responsiveness (CGTCA motif), auxin responsiveness (AuxRR-core, TGA-element), gibberellin responsiveness (Pbox), salicylic acid responsiveness (TCA-element), zein metabolism regulation element (O_2_-site), defense and stress responsiveness (TC-rich repeats), low-temperature responsiveness (LTR), and other elements containing MYB binding sites which regulate light responses and drought stress. A number of elements regulating plant growth and development were also found, including meristem expression element (CAT-box) and endosperm expression element (GCN4-motif). Thus, the members of this family play important roles in the growth and development of *P. yunnanensis*.

### 3.5. Analysis of Codon Preference and Its Influencing Factors

The codon usage bias in the *DET2* family of *P. yunnanensis* was analyzed (Table S3). The codon adaptation index (CAI) of *DET2* family genes ranged from 0.168 to 0.211, with an average of 0.186. The codon preference index (CBI) of *DET2* family genes ranged from −0.142 to 0.045, with an average of −0.05, and the optimal codon usage frequency FOP values ranged from 0.323 to 0.420, with an average of 0.372, and less than 0.5. The content of T3s ranged from 0.3364 to 0.4514, with an average of 0.3967. The content of A3s ranged from 0.1761 to 0.3574, with an average of 0.2851. The content of C3s ranged from 0.1701 to 0.3458, with an average of 0.2674. The content of G3s ranged from 0.2584 to 0.3638, with an average of 0.2803. The frequency of GC1 (except *PyDET2m*), GC2, and GC3(except *PyDET2n*) and the average GC (except *PyDET2n*) content were below 50%.These results indicated a preference for A/U-ending codons.

**Figure 7:**
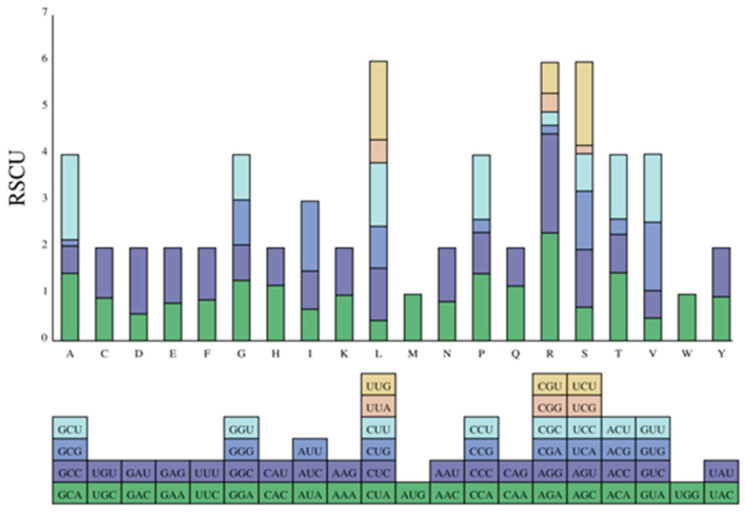
Relative codon usage of the *DET2* gene family in *P. yunnanensis*.

A total of 3933 codons (including stop codons) were found in the 14 *DET2* genes of *P. Yunnanensis*. The RSCU value analysis of the codons showed that there were 2331 codons with RSCU > 1, and the RSCU of AGA was 2.32 with the highest frequency among the 59 codons (excluding stop codon and start codon). There were 29 high-frequency codons that showed obvious A/U preference ending (Figure 7).

**Figure 8:**
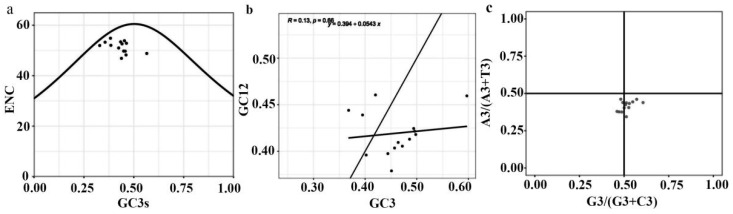
Analysis of influencing factors of preference for the usage of codons in the *DET2* gene family in *P. yunnanensis*. From left to right: (**a**) ENc-plot; (**b**) neutral plot; (**c**): PR2-plot analysis.

The ENc-plot, Neutral-plot, and PR2-plot analyses are shown (Figure 8). The *DET2* family members have GC3s distributions ranging from 0.368 to 0.597, with the exception of *PyDET2n* with a GC3s content of 0.565 (greater than 0.5), and the distribution of CDSs was not evenly around the center point (A = U/T, G = C). So the formation of codon usage patterns is affected by mutation pressure and natural selection. The correlation coefficient and regression coefficient were 0.13 and 0.394, respectively, which indicated that the correlation between the base composition at the first and second position of the codon and that at the third position of the codon was weak, and the coding gene of the *DET2* gene family was highly conserved; codon preference is strongly influenced by selection pressure.

### 3.6. Gene Expression Analysis

**Figure 9:**
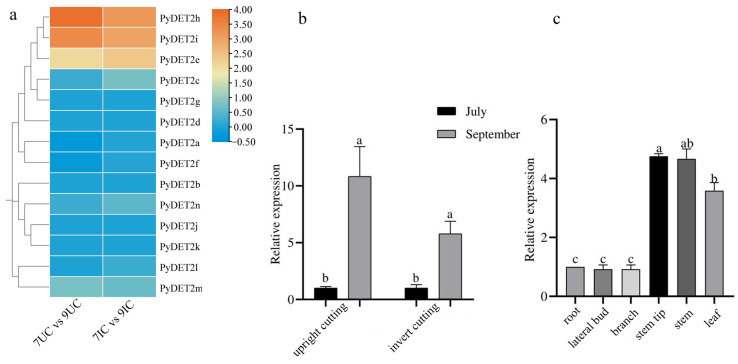
(**a**): The expression patterns of *PyDET2* genes. 7/9UC: upright cuttings of *P. yunnanensis* in July/September. 7/9IC: invert cuttings of *P. yunnanensis* in July/September. Blue or red color indicates lower or higher expression levels of each sample, respectively. (**b**): The relative expression levels of *PyDET2e* in the stem tip in two growth phases and cutting methods. (**c**): The relative expression levels of *PyDET2e* in different tissues. Statistical significance (*p* < 0.05) is indicated by lowercase letters.

Based on the RNA-seq transcriptome data, the expression heatmaps of *DET2* family genes in the stem tips of *P. yunnanensis* at two growth stages (initial and rapid growth stages) and two cuttings (upright cuttings and inverted cuttings) were visualized. The result showed that three genes (*PyDET2h*, *PyDET2i*, and *PyDET2e)* showed higher expression in September than in July, both in inserted and upright cuttings (Figure 9). Therefore, three genes play an important role in the rapid growth of *P. yunnanensis*. We selected *PyDET2e* for qRT-PCR. The results showed that the transcriptome data were reliable. Subsequently, we analyzed its tissue specificity and found that its expression level in the stem, stem tip, and leaf was significantly higher (Figure 9).

### 3.7. Vector Construction and Subcellular Localization of PyDET2e

**Figure 10:**
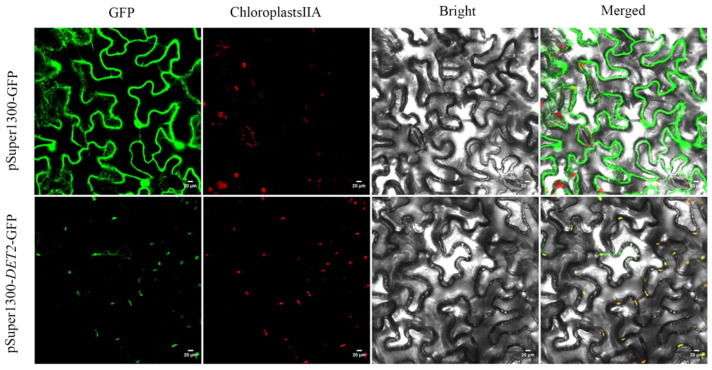
Subcellular localization analysis of *PyDET2e* in the leaves of tobacco; images show an abaxial side view of leaf epidermis with localization of chloroplast marked with chloroplast self-luminescence (Green is *GFP* fluorescence and red is chloroplast self-luminescence).

Online website predictions show that *PyDET2e* is localized to chloroplasts. To determine the location of *PyDET2e* protein in the cells, *PyDET2e*-pSuper1300-*GFP* was used as an experimental group and pSuper1300-*GFP* was used as a control. Three days after the injection of tobacco leaves, the position of the fusion protein was observed by fluorescence confocal microscopy. The results showed that *PyDET2e* protein was distributed in chloroplasts. However, pSuper1300-*GFP* alone was distributed in the cytoplasm and the nucleus (Figure 10).

## 4. Discussion

BRs have a wide range of physiological functions, such as plant height, plant type, flowering time, plant root growth, stem elongation, leaf extension, microtubule system development, plant morphology in dark conditions, pollen tube elongation, and seed development [33,34,35]. The main signal transduction pathways of BRs have been established, and the key gene functions of these pathways have been verified in *A. thaliana*; the *DET2* gene is a key rate-limiting gene in brassinolide biosynthesis pathway, and its effect is self-evident [36,37,38].At present, the *DET2* gene has been identified in many angiosperms and gymnosperms [23,39,40,41,42], and few studies on woody plants have been reported. Some studies on other plants have been limited to the cloning of related homologous genes of *A. thaliana* and the analysis of corresponding mutants. Different plants may have different synthetic pathways, so it is necessary to verify the universality of these pathways or reveal new synthetic pathways through the study of many types of plants in the future. As to whether the BR biosynthesis and signal transduction mechanism of woody plants are identical or specific to *A. thaliana*, and whether there are similarities and differences in the roles played by *DET2*, nothing is certain. The study on the relationship between the steroid dehydrogenase family and its members in woody plants can provide a molecular basis for the rapid growth of *P. yunnanensis* and quicken the process of forest genetic breeding. The whole genome sequencing of *P. yunnanensis* has been completed, which provides important data support for the molecular biology research of *P. yunnanensis*.

Fourteen *DET2* genes were identified in this study. The *PyDET2* proteins have different sequence lengths. Most *PyDET2* proteins have isoelectric points greater than 7, suggesting that *PyDET2* genes may encode an alkaline protein that exerts biological functions in alkaline subcellular environments [43]. The 14 genes of the *DET2* family were classified into four groups (group 1, group 2, group 3, and group 4). The analysis of the 14 promoter regions of *PyDET2* genes suggests that the *PyDET2* proteins are involved in many growth and development processes (Figure 6). Codon analysis showed that genes in this family have a clear A/U preference and that natural selection is the main factor influencing their formation (Figure 8). Gene duplication is one of the major forces that act on gene expansion and ultimately drive biological evolution. In total, *14 DET2* genes have evolved repeatedly from WGD and fragments. The two types of gene duplication also contributed to the amplification of *DET2s*. The collinearity analysis showed that some genes had no collinearity between the *DET2* family and *A. thaliana* and *P. trichocarpa*; these may be *DET2* members with new functions in *P. yunnanensis* (Figure 5).

The stem tip secretes and accumulates various hormones, and through growth, division, and differentiation, the stem elongates unceasingly and forms a stem-related structure. In our previous research, we found that the height growth and stem diameter of *P. yunnanensis* cuttings in September were significantly higher than those in July, whether it was upright or inverted cuttings. Based on the RNA-seq of stem-tip and qRT-PCR in the *DET2* family, we found that the expression of the *PyDET2h*, *PyDET2i*, and *PyDET2e* was significantly higher. Three genes have similar motifs and gene structures, which suggests that they have similar effects on the growth of *P. yunnanensis*. These genes are located in group1, and their codon composition is quite similar, so it is inferred that they play similar biological functions (Figure 9).

The *PyDET2e* was selected to clone and construct an over expression vector containing *GFP* for subcellular localization. The CDS length of *PyDET2e* is 795 bp, encoding 264 amino acids. It is an unstable hydrophobic protein with no signal peptide and three transmembrane regions composed of α-helices, and it was presumed that the protein is a non-secretory membrane protein with a hydrophobic index of 0.34, which accords with the characteristics of membrane localization protein. Protein phosphorylation is at the end of the signaling chain and can be achieved via the phosphorylation of transcription factors for the purpose of regulating genes, and the site predictions indicate that there are 17 sites at which serine may be phosphorylated. It is speculated that the changes in the conformation of *PyDET2e* protein may be regulated by the SER phosphorylation site; whether the predicted phosphorylation sites really exist and what roles they play can be further analyzed via protein phosphorylation modification omics and Western blot methods (Figure S2). The subcellular localization results showed that *PyDET2e* was expressed in the chloroplast and a little in the cytoplasm. To determine which part of the chloroplast it is expressed in, further analysis is needed (Figure 10).

Chloroplasts are the main sites of photosynthesis in plants and are involved in many aspects, such as sulfur and nitrogen assimilation, fatty acid, amino acid, and hormone synthesis [43,44,45]. Plant hormones such as brassinolide, cytokinin, auxin, and gibberellin are regulated by light and control chloroplast growth and development [46,47]. In plant green tissues, a complex network of transcription factors, light, and hormone signals is formed, and regarding *PyDET2e*, its expressed within a specific part of the chloroplast and the role of this gene in chloroplast development and BRs synthesis remains to be further studied and demonstrated. In other studies, *GmDET2a and GmDET2b* genes were widely expressed in the root, leaf, and hypocotyl of soybean [42], and the expression of the *LeDET2* gene was highest in the leaves of tomato [41]. The tissue specificity of our study indicated that *PyDET2e* was highly expressed not only in stem tips and stems, but also in leaves, and this may be due to species variability leading to differences in gene function and thus differences in expression tissues. BR does not require polarity transport; it is evident that BR synthesis is active in three tissues, which suggests that it plays an important role in the leaf, stem, and tip of stem development, and the mechanism of *DET2* at different tissues needs to be further investigated.

## 5. Conclusions

We identified 14 *DET2* family genes in *P. yunnanensis* and analyzed the genetic structure, phylogenetic relationships, cis-regulatory elements, collinearity, subcellular localization, and expression. The result suggested that *DET2* genes might be involved in plant growth, development, and hormone signal transduction. The tissue-specific analysis of *PyDET2e* revealed that *PyDET2e* in September was significantly higher than that in July (both in upright and inverted cutting), and the expression of it in the stem, stem tip, and leaf was significantly higher than that in other parts; subcellular localization showed that *PyDET2e* protein was located in the chloroplast.

## Data Availability

Genomic data and RNA-seq date of *P. yunnanensis* can be obtained by contacting the corresponding author. The data that support the findings of this study are available from the corresponding author upon reasonable request.

## Supplementary Materials

The following supporting information can be downloaded at: https://www.mdpi.com/article/10.3390/genes15020148/s1, Table S1: Primer sequence of qRT-PCR; Table S2: Identification of *DET2* family genes in *P. yunnanensis*; Table S3: Base composition of codons in the *DET2* gene family of *P. yunnanensis*; Figure S1: Prediction of the secondary structure of *PyDET2s* protein; Figure S2: Prediction of phosphorylation sites and transmembrane regions of *PyDET2e* protein.

## References

[1] Hacham Y., Holland N., Butterfield C., Ubeda-Tomas S., Bennett M.J., Chory J., Savaldi-Goldstein S. (2011). Brassinosteroid perception in the epidermis controls root meristem size. Development.

[2] Topping J.F., May V.J., Muskett P.R., Lindsey K. (1997). Mutations in the *HYDRA1* gene of Arabidopsis perturb cell shape and disrupt embryonic and seedling morphogenesis. Development.

[3] Oh M.H., Honey S.H., Tax F.E. (2020). The Control of Cell Expansion, Cell Division, and Vascular Development by Brassinosteroids: A Historical Perspective. Int. J. Mol. Sci..

[4] Mohammadreza A., Rana R.-R. (2018). 24-Epibrassinolide enhanced the quality parameters and phytochemical contents of table grape. J. Appl. Bot. Food Qual..

[5] Barket A., Syed A.H., Shamsul H., Qaiser H., Sangeeta Y., Qazi F., Aqil A. (2008). A role for brassinosteroids in the amelioration of aluminium stress through antioxidant system in mung bean (*Vigna radiata* L. Wilczek). Environ. Exp. Bot..

[6] Manghwar H., Hussain A., Ali Q., Liu F. (2022). Brassinosteroids (BRs) Role in Plant Development and Coping with Different Stresses. Int. J. Mol. Sci..

[7] Tachibana R., Abe S., Marugami M., Yamagami A., Akema R., Ohashi T., Nishida K., Nosaki S., Miyakawa T., Tanokura M. (2024). *BPG4* regulates chloroplast development and homeostasis by suppressing *GLK* transcription factors and involving light and brassinosteroid signaling. Nat. Commun..

[8] Zeng L.L., Song L.Y., Wu X., Ma D.N., Song S.W., Wang X.X., Zheng H.L. (2024). Brassinosteroid enhances salt tolerance via S-nitrosoglutathione reductase and nitric oxide signaling pathway in mangrove *Kandelia obovata*. Plant Cell Environ..

[9] Fujioka S., Takatsuto S., Yoshida S. (2002). An early C-22 oxidation branch in the brassinosteroid biosynthetic pathway. Plant Physiology..

[10] Si J., Sun Y., Wang L.U., Qin Y., Wang C., Wang X. (2016). Functional analyses of *populus* euphratica brassinosteroid biosynthesis enzyme genes *DWF4* (*PeDWF4*) and *CPD* (*PeCPD*) in the regulation of growth and development of *Arabidopsis thaliana*. J. Biosci..

[11] Pei D., Ren Y., Yu W., Zhang P., Dong T., Jia H., Fang J. (2023). The roles of brassinosteroids and methyl jasmonate on postharvest grape by regulating the interaction between *VvDWF4* and *VvTIFY 5A*. Plant Sci..

[12] Gómez-Ocampo G., Crocco C.D., Cascales J., Oklestkova J., Tarkowská D., Strnad M., Mora-Garcia S., Pruneda-Paz J.L., Blazquez M.A., Botto J.F. (2023). *BBX21* integrates brassinosteroid biosynthesis and signalling in the inhibition of hypocotyl growth under shade. Plant Cell Physiol..

[13] Wang D., Hao X., Xu L., Zhao M., Wang C., Yu X., Kong Y., Lu M., Zhou G., Chai G. (2023). Fine-tuning brassinosteroid biosynthesis via 3′UTR-dependent decay of *CPD* mRNA modulates wood formation in *Populus*. J. Integr. Plant Biol..

[14] Wang T., Zheng Y., Tang Q., Zhong S., Su W., Zheng B. (2021). Brassinosteroids inhibit miRNA-mediated translational repression by decreasing AGO_1_ on the endoplasmic reticulum. J. Integr. Plant Biol..

[15] Li Y., Wei K. (2020). Comparative Functional Genomics Analysis of Cytochrome P450 Gene Superfamily in Wheat and Maize. BMC Plant Biol..

[16] Zhang C., He M., Wang S., Chu L., Wang C., Yang N., Ding G., Cai H., Shi L., Xu F. (2021). Boron deficiency-induced root growth inhibition is mediated by brassinosteroid signalling regulation in Arabidopsis. Plant J..

[17] Noguchi T., Fujioka S., Takatsuto S., Sakurai A., Yoshida S., Li J., Chory J. (1999). Arabidopsis *det2* is defective in the conversion of (24R)-24-methylcholest-4-En-3-one to (24R)-24-methyl-5alpha-cholestan-3-one in brassinosteroid biosynthesis. Plant Physiol..

[18] Li J., Biswas M., Chao A., Russell D., Chory J. (1997). Conservation of function between mammalian and plant steroid 5α-reductases. Proc. Natl. Acad. Sci. USA.

[19] Chory J., Nagpal P., Peto C.A. (1991). Phenotypic and genetic analysis of *det2*, a new mutant that affects light-regulated seedling development in Arabidopsis. Plant Cell..

[20] Cheon J., Park S.Y., Schulz B., Choe S. (2010). Arabidopsis brassinosteroid biosynthetic mutant *dwarf7-1* exhibits slower rates of cell division and shoot induction. BMC Plant Biol..

[21] Xie L., Yang C., Wang X. (2011). Brassinosteroids can regulate cellulose biosynthesis by controlling the expression of *CESA* genes in Arabidopsis. J. Exp. Bot..

[22] Szekeres M., Koncz C. (1998). Biochemical and genetic analysis of brassinosteroid metabolism and function in Arabidopsis. Plant Physiol. Biochem..

[23] Luo M., Xiao Y., Li X., Lu X., Deng W., Li D., Hou L., Hu M., Li Y., Pei Y. (2007). *GhDET2*, a steroid 5alpha-reductase, plays an important role in cotton fiber cell initiation and elongation. Plant J..

[24] Wang Y., Hao Y., Guo Y., Shou H., Du J. (2022). *PagDET2* promotes cambium cell division and xylem differentiation in poplar stem. Front. Plant Sci..

[25] Zhou F., Hu B., Li J., Yan H., Liu Q., Zeng B., Fan C. (2024). Exogenous applications of brassinosteroids promote secondary xylem differentiation in Eucalyptus grandis. PeerJ..

[26] Liu G., Li Y., Liu Y., Guo H., Guo J. (2021). Genome-wide identification and analysis of monolignol biosynthesis genes in *Salix matsudana Koidz* and their relationship to accelerated growth. For. Res..

[27] Li X., Yang Y., Sun X., Lin H., Chen J., Ren J., Hu X., Yang Y. (2014). Comparative Physiological and Proteomic Analyses of Poplar (*Populus yunnanensis*) Plantlets Exposed to High Temperature and Drought. PLoS ONE.

[28] Luo J.X., Zhen W., Gu Y.J., Cao X.J. (2006). Study on the growth characteristics of *Populus yunnanensis*. J. Southwest For. Univ..

[29] Liu Y.Q., Fu D.R. (2004). Development and Utilization of Sect III. Tacamachaca Gene Resources on the Plateau of Western Sichuan. J. Cent. South. For. Univ..

[30] Chen C., Wu Y., Li J., Wang X., Zeng Z., Xu J., Liu Y., Feng J., Chen H., He Y. (2023). TBtools-II: A “one for all, all for one” bioinformatics platform for biological big-data mining. Mol. Plant..

[31] Sudhir K., Glen S., Li M., Christina K., Koichiro T. (2018). Mega X: Molecular evolutionary genetics analysis across computing platforms. Mol. Biol. Evol..

[32] R Core Team (2018). R: A Language and Environment for Statistical Computing.

[33] Anwar A., Liu Y., Dong R., Bai L., Yu X., Li Y. (2018). The physiological and molecular mechanism of brassinosteroid in response to stress: A review. Biol. Res..

[34] Yang Y., Chu C., Qian Q., Tong H. (2024). Leveraging brassinosteroids towards the next Green Revolution. Trends Plant Sci..

[35] Bajguz A., Piotrowska-Niczyporuk A. (2023). Biosynthetic Pathways of Hormones in Plants. Metabolites..

[36] Aitken V., Diaz K., Soto M., Olea A.F., Cuellar M.A., Nuñez M., Espinoza-Catalán L. (2023). New Brassinosteroid Analogs with 23,24-Dinorcholan Side Chain, and Benzoate Function at C-22: Synthesis, Assessment of Bioactivity on Plant Growth, and Molecular Docking Study. Int. J. Mol. Sci..

[37] Wang Z.Y., Bai M.Y., Oh E., Zhu J.Y. (2012). Brassinosteroid signaling network and regulation of photomorphogenesis. Annu. Rev. Genet..

[38] Wang Z.Y., Nakano T., Gendron J., He J., Chen M., Vafeados D., Yang Y., Fujioka S., Yoshida S., Asami T. (2002). Nuclear-localized *BZR1* mediates brassinosteroid-induced growth and feedback suppression of brassinosteroid biosynthesis. Dev. Cell..

[39] Kutschera U., Wang Z.Y. (2012). Brassinosteroid action in flowering plants: A Darwinian perspective. J. Exp. Bot..

[40] Hayat S., Ahmad A. (2011). Brassinosteroids: A Class of Plant Hormone.

[41] Rosati F., Bardazzi I., De Blasi P., Simi L., Scarpi D., Guarna A., Serio M., Racchi M.L., Danza G. (2005). 5alpha-Reductase activity in Lycopersicon esculentum: Cloning and functional characterization of LeDET2 and evidence of the prealsence of two isoenzymes. J. Steroid Biochem. Mol. Biol..

[42] Huo W., Li B., Kuang J., He P., Xu Z., Wang J. (2018). Functional Characterization of the Steroid Reductase Genes *GmDET2a* and *GmDET2b* form *Glycine max*. Int. J. Mol. Sci..

[43] Cackett L., Luginbuehl L.H., Schreier T.B., Lopez-Juez E., Hibberd J.M. (2022). Chloroplast development in green plant tissues: The interplay between light, hormone, and transcriptional regulation. New Phytol..

[44] Zhang D., Tan W., Yang F., Han Q., Deng X., Guo H., Liu B., Yin Y., Lin H. (2021). A BIN2-GLK1 Signaling Module Integrates Brassinosteroid and Light Signaling to Repress Chloroplast Development in the Dark. Dev. Cell..

[45] Gutkowska M., Buszewicz D., Zajbt-Łuczniewska M., Radkiewicz M., Nowakowska J., Swiezewska E., Surmacz L. (2023). Medium-chain-length polyprenol (C45-C55) formation in chloroplasts of Arabidopsis is brassinosteroid-dependent. J. Plant Physiol..

[46] Waadt R., Seller C.A., Hsu P.-K., Takahashi Y., Munemasa S., Schroeder J.I. (2022). Plant hormone regulation of abiotic stress responses. Nat. Rev. Mol. Cell Biol..

[47] Lyons G., Carmichael E., Mcroberts C., Aubry A., Thomson A., Reynolds C.K. (2018). Prediction of lignin content in ruminant dietsand fecal samples using rapid analytical techniques. J. Agric. Food Chem..

