# Supplementary material for: Genome-Wide Identification, Expression Analysis, and Subcellular Localization of DET2 Gene Family in Populus yunnanensis
Source: Genes (Basel). 2024 Jan 23;15(2):148. doi: 10.3390/genes15020148 (PMC10888042; doi:10.3390/genes15020148)
Supplement: Supplementary file 1 [file genes-15-00148-s001.zip › genes-2792049-supplementary.pdf]

Table S1 Primer sequence of RT-qPCR

Gene name	Forward primer (5′→3′)	Reverse primer (5′→3′)
PyDET2e	TACAAGGTTCCCAAGGGTGG	AACTCCTCCCCGTCAGGTAA
HIS	TTTAAGACTGATCTGCGTTTCC	GAACAGCCCAACAAGGTATG

Table S2. Identification of *DET2* family genes in *P. yunnanensis.*

Gene name	Gene ID	Amino Acid(aa)	Molecular Weight (kDa)	Isoelectric Point (pI)	Instability Index	GRAVY	Secondary structure/%				Subcellular Localization
							Alpha helix	Extended strand	Beta turn	Random coil	
PyDET2a	*Pyun04G015560.1*	339	38.94	8.79	46.84	102.36	53.98	16.22	3.83	25.96	Chloroplast
PyDET2b	*Pyun05G003670.1*	253	29.71	9.17	38.71	86.68	29.25	28.46	4.74	37.55	Golgi apparatus
PyDET2c	*Pyun08G000950.1*	267	30.10	9.65	42.71	110.90	51.69	17.23	2.62	28.46	Chloroplast.<br>Cell membrane
PyDET2d	*Pyun08G000960.1*	263	29.62	9.53	31.45	102.59	49.43	17.11	5.32	28.14	Chloroplast<br>Golgi apparatus
PyDET2e	*Pyun08G000970.1*	264	30.02	9.66	43.18	108.11	47.35	21.97	3.79	26.89	Chloroplast
PyDET2f	*Pyun09G012590.1*	351	40.35	8.99	44.34	101.11	47.29	19.66	4.84	28.21	Cell membrane
PyDET2g	*Pyun10G023000.1*	268	30.26	9.40	45.98	109.78	48.51	17.91	4.85	28.73	Cell membrane<br>Chloroplast<br>Peroxisome
PyDET2h	*Pyun10G023010.1*	253	28.74	9.58	40.06	106.32	48.22	18.58	4.74	28.46	Cell membrane
PyDET2i	*Pyun10G023020.1*	264	29.76	9.34	43.02	107.08	54.20	13.74	4.2	27.86	Cell membrane<br>Golgi apparatus<br>Peroxisome
PyDET2j	*Pyun13G013760.1*	264	30.90	9.35	43.38	89.02	34.09	25.38	3.41	37.12	Cell membrane<br>Golgi apparatus<br>Mitochondrion
PyDET2k	*Pyun13G013800.1*	264	20.71	9.35	45.08	89.39	36.74	24.24	2.65	36.36	Cell membrane<br>Golgi apparatus<br>Mitochondrion
PyDET2l	*Pyun14G008550.1*	320	38.25	9.25	22.63	94.69	43.12	20.62	3.12	33.12	Chloroplast

PyDET2m	*Pyun15G004230.1*	292	33.11	8.99	28.32	114.79	46.58	17.47	4.45	31.51	Cell membrane
PyDET2n	*Pyun16G010440.1*	267	29.68	9.48	46.55	91.44	38.52	24.51	3.50	33.46	Cell membrane

Note: Information provided includes gene names, gene ids, amino acid, molecular weight (kDa), isoelectric points(PI), instability index, GRAVY, Secondary structure and subcellular localization.

Table S3. Base composition of codons in the *DET2* gene family of *P. yunnanensis.*

Gene Name	T3s/%	C3s/%	A3s/%	G3s/%	CAI/%	CBI/%	Fop/%	ENc/%	GC1/%	GC2/%	GC3/%	GC/%
PyDET2a	0.4514	0.1701	0.3529	0.2617	0.193	-0.120	0.335	51.96	0.482	0.410	0.368	0.419
PyDET2b	0.4135	0.2933	0.2485	0.2785	0.172	-0.142	0.323	53.86	0.450	0.394	0.496	0.448
PyDET2c	0.3930	0.2620	0.2900	0.2707	0.191	-0.002	0.397	50.99	0.429	0.366	0.444	0.413
PyDET2d	0.3991	0.2790	0.2698	0.2798	0.189	-0.011	0.402	46.88	0.383	0.375	0.451	0.403
PyDET2e	0.3689	0.2800	0.2814	0.3149	0.184	-0.055	0.367	52.85	0.460	0.351	0.472	0.429
PyDET2f	0.4184	0.1973	0.3574	0.2622	0.184	-0.078	0.362	53.27	0.463	0.415	0.395	0.425
PyDET2g	0.4353	0.2328	0.2965	0.2584	0.199	-0.023	0.388	52.03	0.424	0.368	0.402	0.398
PyDET2h	0.3641	0.2903	0.3118	0.2695	0.168	-0.096	0.343	52.62	0.449	0.358	0.457	0.423
PyDET2i	0.3722	0.2870	0.2915	0.2787	0.176	-0.063	0.363	49.75	0.453	0.366	0.462	0.428
PyDET2j	0.4045	0.3091	0.2443	0.2761	0.181	-0.058	0.371	48.22	0.430	0.404	0.498	0.446
PyDET2k	0.4045	0.3091	0.2472	0.2635	0.188	-0.032	0.388	49.71	0.438	0.411	0.494	0.449
PyDET2l	0.3900	0.2741	0.3029	0.2880	0.191	-0.063	0.369	53.41	0.433	0.393	0.486	0.438
PyDET2m	0.4033	0.2140	0.3213	0.2598	0.211	0.045	0.420	54.85	0.515	0.406	0.420	0.447
PyDET2n	0.3364	0.3458	0.1761	0.3636	0.180	-0.043	0.376	48.81	0.481	0.438	0.600	0.506

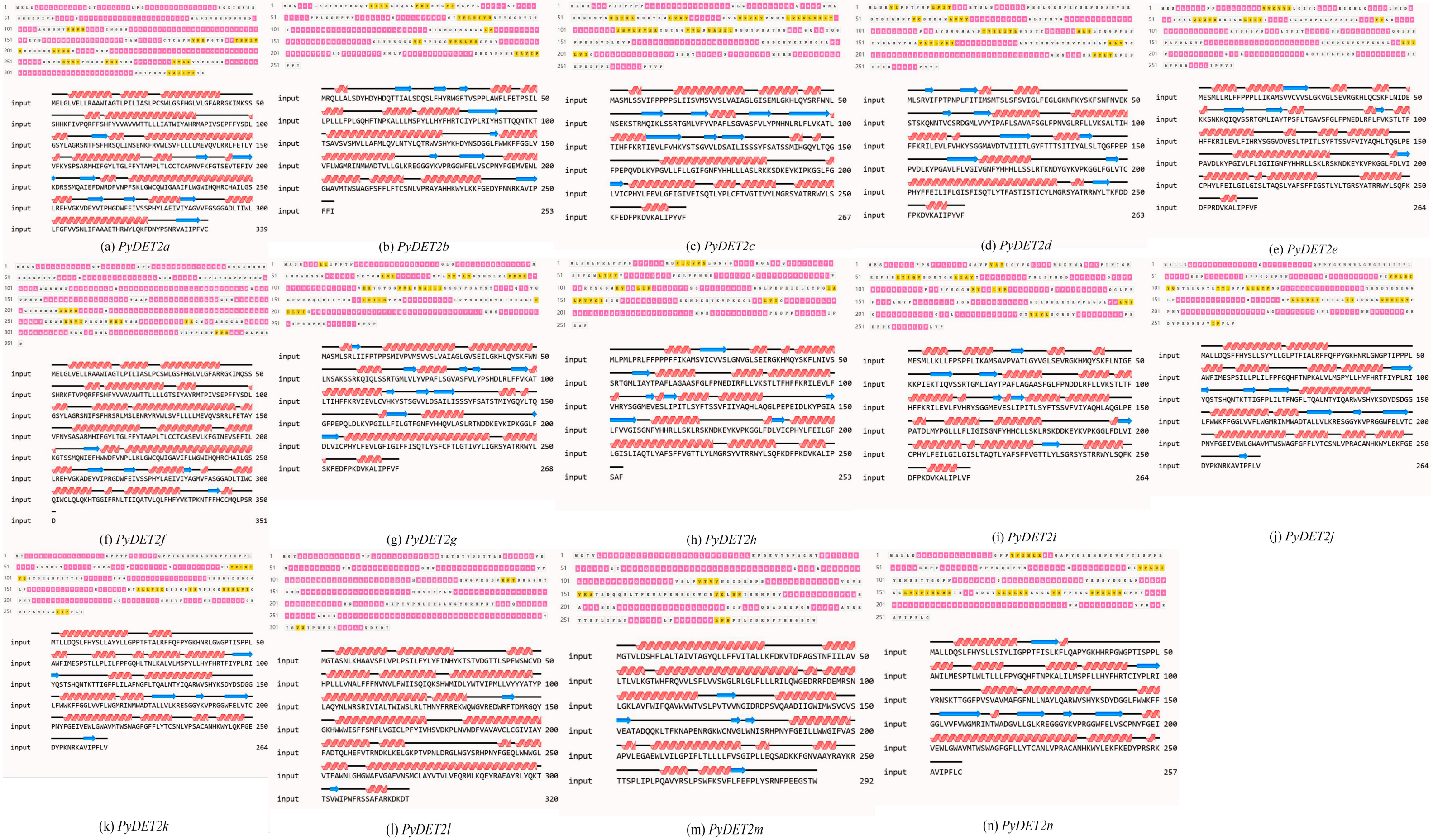

Figure S1. Prediction of the secondary structure of *PyDET2s* protein

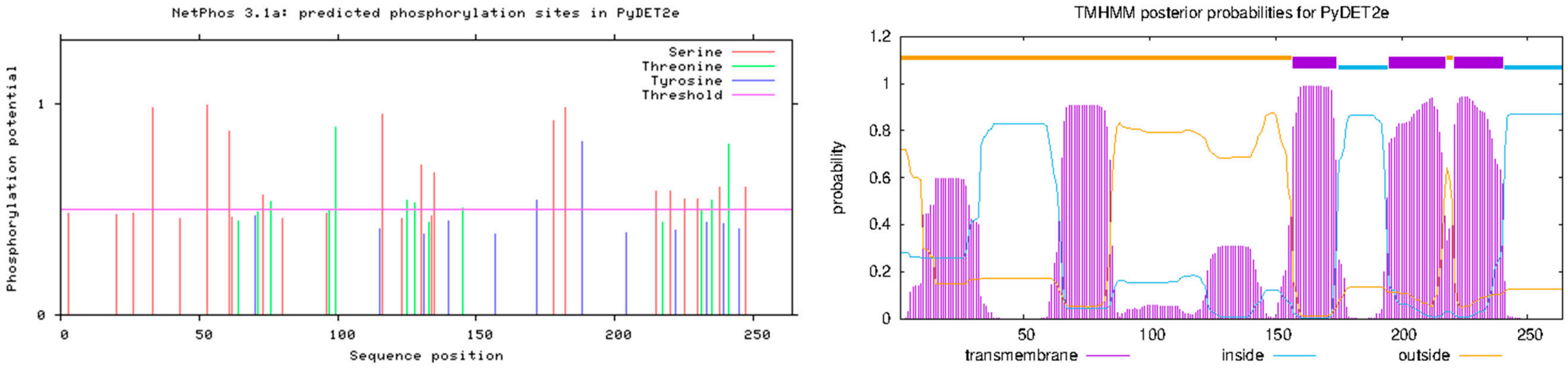

Figure S2. Prediction of phosphorylation sites and transmembrane regions of *PyDET2e* protein
